# Performance of a Novel Computational Hyperemic Resistance Index Derived from Cardiac CT in Coronary Chronic Syndromes

**DOI:** 10.3390/jcm14207270

**Published:** 2025-10-15

**Authors:** Yahia Bellouche, Clement Benic, Sinda Hannachi, Pierre Phillipe Nicol, Christopher Jousse, Florent Le Ven, Jacques Mansourati, Bastien Pasdeloup, Romain Didier

**Affiliations:** 1 Cardiology Department, Brest University Hospital, 29200 Brest, France; 2 Inserm UMR 1304 (GETBO), Western Brittany University Brest, 29200 Brest, France; 3 Lab-STICC, UMR CNRS 6285, IMT Atlantique, 29238 Brest, France

**Keywords:** computational fluid dynamics (CFD), coronary computed tomography angiography (CCTA), hyperemic stenosis resistance (HSR), fractional flow reserve (FFR), chronic coronary syndrome (CCS), wall shear stress (WSS)

## Abstract

**Background/Objectives:** Coronary artery disease (CAD) remains the leading global cause of mortality, underscoring the need for functional assessments that extend beyond anatomical evaluation. The Hyperemic Stenosis Resistance (HSR) index combines invasive pressure and flow parameters to assess stenosis severity but faces limitations due to methodological and standardization challenges. This study aimed to introduce and validate a novel non-invasive computational equivalent of HSR (*cHSR*), derived from coronary computed tomography angiography (CCTA), and to compare its diagnostic performance with fractional flow reserve derived from computational fluid dynamics (FFR_CFD_) and quantitative flow ratio (QFR). **Methods:** A retrospective analysis was conducted on 64 patients (106 coronary lesions) with suspected chronic coronary syndrome (CCS) who underwent both CCTA and invasive coronary angiography (ICA). Computational simulations incorporated patient-specific boundary conditions based on CCTA-derived left ventricular and aortic flow data. Diagnostic accuracy for predicting revascularization was compared among *cHSR*, FFR_CFD_, and QFR. **Results**: FFR_CFD_ showed a strong correlation with invasive FFR (r = 0.87, *p* < 0.0001). The *cHSR* index achieved the highest diagnostic accuracy (96.2%) at an optimal cut-off of 0.75 mmHg/cm·s^−1^, outperforming both FFR_CFD_ and QFR. No significant correlation was found between *cHSR* and shear stress parameters, including the Oscillatory Shear Index (*OSI*) and Time-Averaged Wall Shear Stress (TAWSS), indicating complex hemodynamic interactions beyond simple flow–pressure relationships. **Conclusions**: The computational hyperemic stenosis resistance (*cHSR*) index represents a promising non-invasive tool for the functional assessment of CAD, demonstrating superior diagnostic performance compared with existing imaging-based indices. Prospective multicenter studies with larger populations are warranted to confirm its clinical applicability and prognostic value in chronic coronary syndrome management.

## 1. Introduction

Coronary artery disease (CAD) is the primary global cause of mortality, accounting for 9 million deaths annually, or roughly one in seven deaths worldwide, according to the World Health Organization (WHO). While invasive coronary angiography (ICA) and quantitative coronary angiography (QCA) traditionally assess the anatomical severity of stenosis, relying solely on these measures often proves insufficient. In different series, 20–30% of lesions deemed severe by QCA do not induce ischemia during functional testing [1]. This discrepancy highlights the crucial need for integrating functional evaluations into the revascularization decision-making process to improve patient outcomes [2].

Fractional Flow Reserve (FFR) has emerged as the gold standard for functional lesion assessment and is strongly predictive of adverse cardiovascular events [3,4,5]. Despite its robustness, FFR does not provide information about the microvascular function, with a known discrepancy between FFR and Coronary Flow Reserve (CFR) in up to 20% cases in both normal and altered CFR populations [6]. While FFR assesses pressure gradients across stenosis under hyperemia, microvascular resistance is neutralized by hyperemic agents (adenosine mainly). However, cases of microvascular dysfunction can cause ischemia independently of epicardial stenosis [7,8]. To address these gaps, the Hyperemic Stenosis Resistance (HSR) index was introduced, combining pressure and flow data to offer a more refined physiological assessment [9]. However, the lack of standardized methods for flow measurement has limited its routine clinical use [10]. In a recent study conducted by Boerhout and al., using data from 853 patients and 1107 vessels from the multicentric ILIAS Registry, they aimed to assess HSR’s diagnostic and prognostic value in chronic coronary syndrome (CCS) patients. HSR more accurately identified inducible ischemia compared to FFR and CFR (AUC 0.71 vs. 0.66 and 0.62, respectively; *p* < 0.005). Additionally, abnormal HSR was a strong, independent predictor of target vessel failure at 5-year follow-up (HR 3.80, 95% CI: 2.12–6.73; *p* < 0.005). In cases where revascularization was deferred, HSR showed superior ability in pinpointing vessels that might still benefit from intervention [11].

In the quest for non-invasive diagnostic alternatives, functional imaging techniques like stress SPECT, echocardiography, and cardiac magnetic resonance (CMR) are commonly used to evaluate ischemia [2]. However, their diagnostic accuracy can be limited in high-risk populations, such as patients with left main or three-vessel disease, where anatomical assessment is crucial for risk stratification [12]. In such cases, combining functional and anatomical data is necessary and may provide a more comprehensive evaluation.

Recent advancements in computational modeling, based on Computational Fluid Dynamics (CFD), have enhanced the diagnostic utility of anatomical imaging by simulating coronary flow and pressure [13]. Quantitative Flow Ratio (QFR), based on ICA, and Computed Tomography Fractional Flow Reserve (FFRCT), based on coronary computed tomography angiography (CCTA), both use CFD models to estimate the functional significance of coronary stenoses [14,15]. These techniques have demonstrated improved diagnostic accuracy by offering both anatomical and functional insights with an acceptable cost-effectiveness ratio [16]. However, the performance of a model relies heavily on the defined boundary conditions (BCs). Most available commercial software uses theoretical assumptions and idealized parameters, which can miss the patient-specific setting. When clinical data are accessible, using patient-specific BCs is preferable over generalized or estimated parameters, which produce more realistic flow/pressure curves, particularly near side branches and curvatures where atherosclerotic plaques commonly form [17].

This study proposes a new non-invasive computational equivalent of Hyperemic Stenosis Resistance (*cHSR*) index derived from CCTA to provide a combined flow/pressure-based assessment of coronary lesions. We aim to evaluate its diagnostic performance in predicting revascularization, with FFR_CFD_ and QFR serving as comparators. Furthermore, we explored the relationship between *cHSR* and shear stress parameters, such as Oscillatory Shear Index (*OSI*) and Time-Averaged Wall Shear Stress (TAWSS).

## 2. Methods

### 2.1. Population of Study and Inclusion/Exclusion Criteria

**Figure 1:**
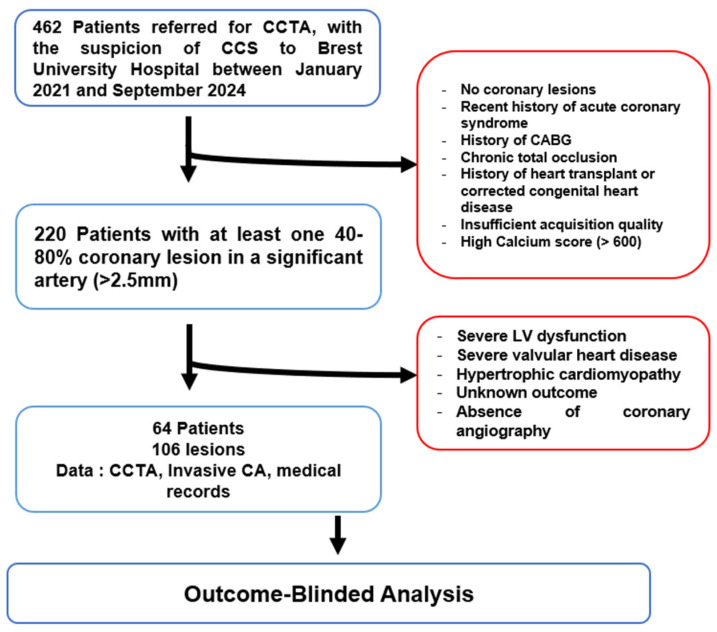
Study flowchart CCTA: Coronary Computed Tomography Angiography; CCS: ChronicCoronary Syndrome; CABG: Coronary Artery Bypass Grafting; LV: Left Ventricular; CA: Coronary Angiography.

This retrospective observational outcome-blinded study was conducted at Brest University Hospital between January 2021 and September 2024. Patients referred for CCTA due to suspected chronic coronary syndrome (CCS), who subsequently underwent ICA, were included. The local Institutional Ethics Committee approved the study protocol, which was based on Reference Methodology (MR004), as defined by the French Data Protection Authority (CNIL), and patient data were anonymized in compliance with institutional regulations (Figure 1).

Inclusion criteria were patients aged between 18 and 85 years who underwent both CCTA and ICA, with at least one significant lesion (40–80%) in a coronary artery larger than 2.5 mm in diameter. Patients were excluded if they had a recent acute coronary syndrome (<40 days), a left ventricular ejection fraction (LVEF) < 40%, significant mitral or aortic valve disease, prior coronary artery bypass graft surgery (CABG), heart transplant, or the presence of chronic total occlusion (CTO). Sixty-four patients with 106 coronary lesions were included in the analysis; the final revascularization decision was omitted from the analysts throughout the study protocol.

In our cohort, the decision whether to proceed with revascularization or not was informed by various methods. Invasive fractional flow reserve (FFR) guided 60% (*n* = 63 lesions) of the revascularization decisions. An additional 30% (*n* = 32 lesions) of the decisions were based on stress-induced ischemia detected by stress single-photon emission computed tomography (SPECT), while in the remaining 10% (*n* = 11 lesions) of patients, revascularization was indicated by electrocardiographic changes and the presence of wall motion abnormalities on rest imaging. Clinical coronary disease pretest probability and coronary calcium scores were calculated in accordance with the most recent guidelines [2].

### 2.2. Data Acquisition

**Figure 2:**
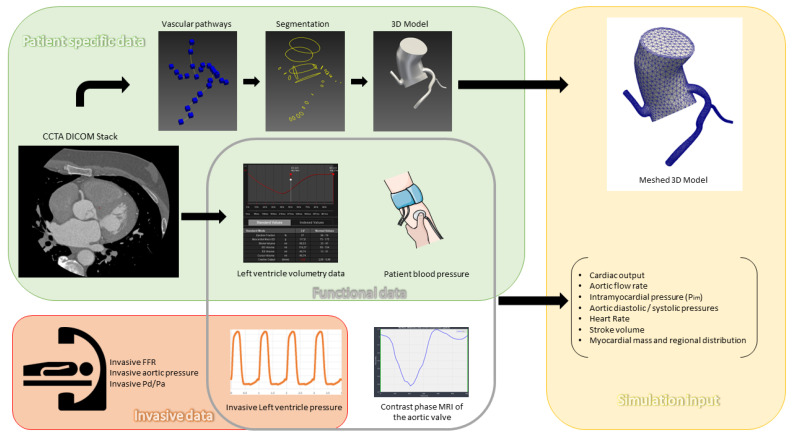
Patient data preprocessing: CCTA: Coronary Computed Tomography Angiography; DICOM: Digital; Imaging and Communications in Medicine; FFR: Fractional Flow Reserve; Pa: Aortic Pressure; Pd: Distal Coronary Pressure; MRI: Magnetic Resonance Imaging.

CCTA was performed using a 320-slice volumetric CT scanner (Aquilion One, Toshiba, Tochigi City, Japan) with retrospective ECG-gating and tube modulation. Image acquisition parameters included a slice thickness of 0.6 mm, with a non-ionic contrast agent injected at 5 mL/s followed by a saline flush. The images were reconstructed using the open source Slicer 3D software (version 5.3.0); segmentation and meshing of coronary artery geometries were conducted with SimVascular (version may 2023), an open-source cardiovascular modeling platform [18]. For each patient, we calculated total left ventricular myocardial mass and allocated percentages for each artery through visual analysis. Invasive blood pressure waveforms (which can be replaced by non-invasive blood pressure), stroke volume, and systolic ejection time were important input data for the sake of individualized flow and pressure targets in the parameters tuning process (Figure 2).

In addition, we modeled aortic flow rates using phase contrast cardiac MRI from 15 healthy individuals, adjusted according to each patient’s body surface area (BSA). For intramyocardial pressure modeling, we acquired left ventricular pressure measurements from 22 healthy individuals, also scaled by BSA, to simulate patient-specific myocardial loading conditions more accurately.

ICA was performed following standard clinical protocols, and invasive FFR measurements were obtained using the PressureWire X (Abbott Vascular, Santa Clara, CA, USA). Hyperemia was induced using intravenous adenosine at 140 μg/kg/min besides intracoronary nitroglycerin to avoid spastic coronary lesions. For the purpose of internal model validation, we collected pressure waveforms of the 60% of lesions that had invasive FFR measurements. Quantitative Flow Ratio (QFR) was derived from ICA images through QAngio XA 3D software (research edition, version 2.1, Medis, Leiden, The Netherlands).

### 2.3. Simulations Pipeline and Measurements

Our simulation pipeline used a patient-specific model (which consisted of the initial aorta, and the three coronary arteries) obtained from CCTA, with segmentation and meshing performed in SimVascular platform (as detailed in Supplementary Materials) [18]. Aortic inflow boundary conditions were calculated based on CCTA-derived left ventricular volumetric data to produce a patient-specific aortic flow rate curve (which guided scaling of curves obtained in MRI to adapt to each patient’s hemodynamics and body surface area). Invasive aortic and intraventricular pressure measurements taken during the ICA served both to calculate initial resistances and as targets for lumped parameters tuning.

Outlet boundary conditions of the peripheral arterial system were based on a simple three-element Windkessel model containing resistances (R_p_ for proximal arteries and R_d_ for distale and microvascular systems) and a capacitator (aortic compliance) [19]. Coronary outlet boundary conditions were adapted from Mantero et al. [20] and modified to exclude coronary venous microcirculation compliance for computational simplicity while preserving realistic coronary flow and pressure dynamics. This coronary vascular model incorporated arterial resistance (R_a_) and compliance (C_a_), arterial microcirculation resistance (R_a-micro_), myocardial compliance (C_im_), venous microcirculation resistance (R_v-micro),_ and intramyocardial pressure (P_im_) to achieve patient-specific hemodynamic responses.

**Figure 3:**
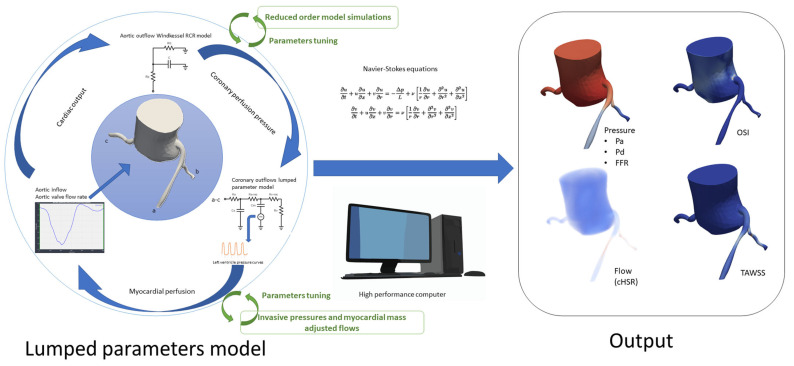
Simulation process and output: Pa: Aortic Pressure; Pd: Distal Coronary Pressure; FFR: Fractional Flow Reserve; *cHSR*: Computational Hyperemic Stenosis Resistance; *OSI*: Oscillatory Shear Index; TAWSS: Time-Averaged Wall Shear Stress; RCR Model: Resistance, Capacitance, and Resistance model (used in Windkessel outflow modeling); Navier-Stokes Equations: Fundamental equations for fluid dynamics, used to model blood flow.

**Figure 4:**
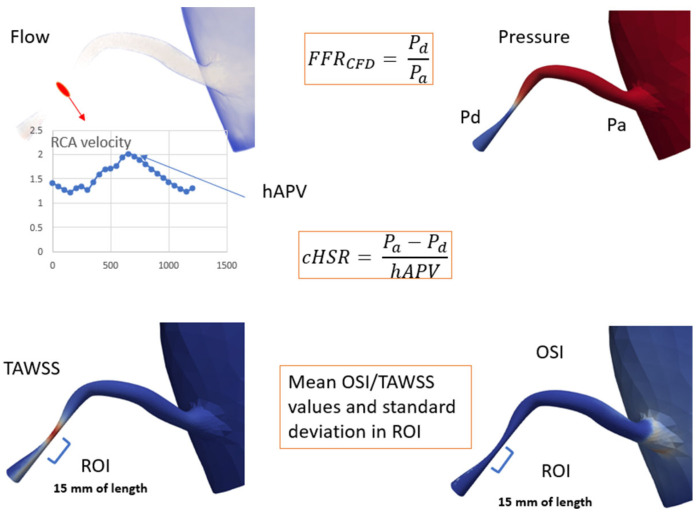
Computational analysis illustrating key parameters: FFR_CFD_: Fractional Flow Reserve via Computational Fluid Dynamics; hAPV: Hyperemic Average Peak Velocity; *cHSR*: Computational Hemodynamic Stenosis Resistance; Pd: Distal Pressure, Pa: Proximal Pressure; TAWSS: Time-Averaged Wall Shear Stress; *OSI*: Oscillatory Shear Index; ROI: Region of Interest, 15 mm of vessel length beyond peak stenosis.

To optimize computational accuracy, both models’ lumped parameters were refined using reduced-order simulations, and different conditions (Rest and exercise) were tested according to the methods proposed by kim and al. [21]. Initial values and tunning process are detailed in Supplementary Materials. Tridimensional CFD simulations were executed after that, using the SimVascular gateway [22], solving Navier-Stokes equations to compute hemodynamic parameters, including pressure, velocity, wall shear stress (WSS), and flow at each point of the coronary arteries tree. These simulations were performed under both basal and hyperemic conditions and produced output, allowing for assessment of stenosis severity in relation to functional coronary flow (Figure 3 and Figure 4). *FFR_CFD_* was calculated as the ratio of distal coronary pressure *P_dist_* to aortic pressure *P_aortic_* during hyperemia:

$${FFR}_{CFD}=\frac{P_{dist}}{P_{aortic}}$$

Similarly, *cHSR* was calculated by dividing the pressure gradient across the lesion (Δ*P*) by the hyperemic average peak velocity (hAPV) as measured at stenosis level through 3D CFD simulation results:

$$cHSR=\frac{∆P}{hAPV}$$

Wall shear stress (WSS) quantifies the tangential force exerted by blood flow on the vessel wall and plays a critical role in endothelial function and atherosclerosis progression. Time-Averaged Wall Shear Stress (TAWSS) was computed using the equation:

$$TAWSS=\;\frac{1}{T}\int_{0}^{T}\left|\tau (t)\right|dt$$

where $\tau \left(t\right)$ represents the instantaneous WSS at a given point on the arterial wall, and *T* is the cardiac cycle duration. This provides an overall measure of shear stress magnitude experienced by the vessel wall over the cardiac cycle.

The Oscillatory Shear Index (*OSI*) Quantifies the Directional Variability of WSS and Is Calculated as

$$OSI=\frac{1}{2}(1-\frac{\left|\int_{0}^{T}\tau \left(t\right)dt\right|}{\int_{0}^{T}\left|\tau (t)\right|dt})$$

where the numerator accounts for the net directional shear stress over a full cycle, while the denominator represents the total shear magnitude. *OSI* values close to 0 indicate unidirectional flow, whereas values approaching 0.5 suggest significant oscillations in shear direction. Both TAWSS and *OSI* were computed from CFD simulations using Navier-Stokes equations under pulsatile flow conditions, with coronary flow profiles extracted from patient-specific CCTA-derived aortic inflow and coronary geometry. These parameters were analyzed at regions of interest (15 mm beyond the peak stenosis) to evaluate their relationship with *cHSR* and lesion severity.

### 2.4. Statistical Analysis

For statistical analysis in our study, we used R Statistical Software (v4.4.1; R Core Team 2021). Descriptive statistics were used to summarize the baseline characteristics of the study population and coronary lesions. Normally distributed continuous variables were presented as mean and standard deviation (SD), while non-normally distributed variables were expressed as median and interquartile range (IQR). The Shapiro-Wilk test was used to assess normality, and categorical variables were described as counts and percentages.

The agreement between FFR_CFD_ and invasive FFR was assessed using a Bland-Altman plot, and their correlation was evaluated using Pearson’s correlation coefficient. The optimal cut-off for *cHSR* was determined using Youden’s index. Diagnostic performance of *cHSR* for predicting revascularization was assessed using ROC curve analysis, with area under the curve (AUC), sensitivity, specificity, positive predictive value (PPV), and negative predictive value (NPV) calculated for each method. All these metrics were compared to those of FFR_CFD_ and QFR using the DeLong test.

Correlation between *cHSR* and wall shear stress parameters (TAWSS, *OSI*) was evaluated using Pearson’s correlation coefficient. Statistical significance was set at *p* < 0.05.

## 3. Results

### 3.1. Patient Population and Lesion Characteristics

A total of 64 patients (mean age 67.8 ± 9.2 years) were included in the study, with a pre-test probability for coronary artery disease (CAD) averaging 24.5 ± 14.8%. The cohort had a sex ratio of 3:1. Among the patients, 62% had hypertension (HTN), 39% had diabetes mellitus (DM), 41% had a history of smoking, and 40% had dyslipidemia. Obesity was noted in 34% of the cohort, and 20% had chronic kidney disease (CKD). The mean coronary artery calcium (CAC) score was 113.1 ± 102.4 Agatston units (Table 1).

In terms of coronary lesions, a total of 106 lesions were analyzed, with a mean stenosis length of 18.0 ± 7.4 mm. The distribution of lesions across different coronary arteries was as follows: 49% in the left anterior descending artery (LAD), 26% in the left circumflex artery (LCx), 22% in the right coronary artery (RCA), and 3% in the left main artery (LM). The mean stenosis percentage, as determined by quantitative coronary angiography (QCA), was 55.3 ± 11.2% (Table 2).

### 3.2. Correlation Between FFR_CFD_ and Invasive FFR

A Bland-Altman analysis was used to assess the agreement between FFR_CFD_ and invasive FFR, revealing good agreement with a Pearson correlation coefficient of 0.87 (*p* < 0.0001), indicating strong linear correlation. The plot confirmed minimal bias (mean difference + 0.01), supporting the validity of the computational FFR_CFD_ measurements.

### 3.3. cHSR Cut-Off Determination

The optimal cut-off value for *cHSR* to predict the need for revascularization was determined through receiver operating characteristic (ROC) curve analysis and the Youden Index. The ideal *cHSR* cut-off was found to be 0.75 mmHg/cm·s^−1^, providing the highest overall diagnostic accuracy. At this cut-off, the sensitivity and specificity for predicting revascularization were 96.2% each.

### 3.4. Diagnostic Performance of cHSR, FFR_CFD_, and QFR

**Figure 5:**
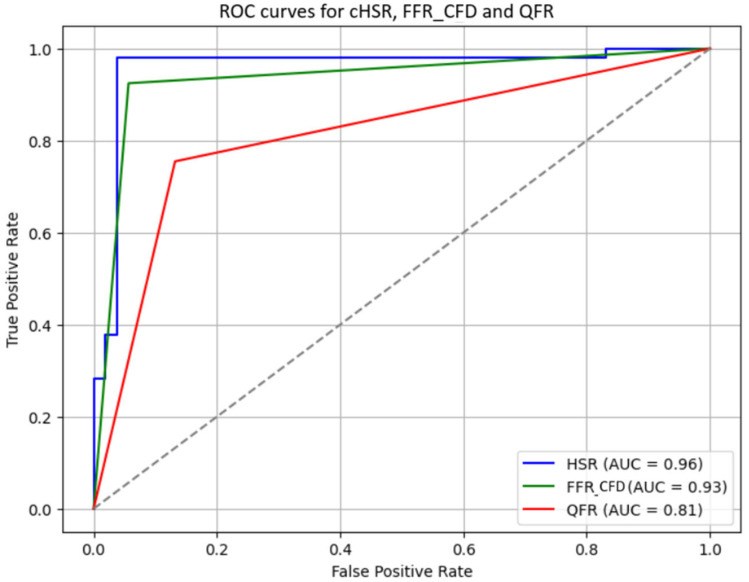
Receiver operator curves for *cHSR*, FFRCFD, and QFR: *cHSR*: Computational Hyperemic Stenosis Resistance; FFR_CFD_; Computed Fractional Flow Reserve; QFR: Quantitative Flow Ratio; AUC: Area Under the Curve; ROC: Receiver Operating Characteristic.

The diagnostic performance of *cHSR*, FFR_CFD_, and QFR for predicting the necessity of revascularization was compared (Figure 5). *cHSR* demonstrated the best diagnostic accuracy (96.2%) compared to FFR_CFD_ (83.0%) and QFR (81.1%). *cHSR* also showed superior sensitivity and specificity (96.2%) when compared to FFR_CFD_ (sensitivity 71.7%, specificity 94.3%) and QFR (sensitivity 75.5%, specificity 86.8%). The positive predictive value (PPV) and negative predictive value (NPV) for *cHSR* were also both 96.6%, significantly higher than those of FFR_CFD_ and QFR (Table 3).

### 3.5. DeLong Test Results

The DeLong test was used to statistically compare the areas under the ROC curves (AUCs) of *cHSR*, FFR_CFD_, and QFR. The results showed that *cHSR* had a significantly higher AUC compared to both FFR_CFD_ (z = 19.27, *p* < 0.001) and QFR (z = 16.55, *p* < 0.001), further confirming its superior diagnostic performance in predicting the necessity of revascularization.

### 3.6. Correlation Between cHSR, TAWSS, and OSI

**Figure 6:**
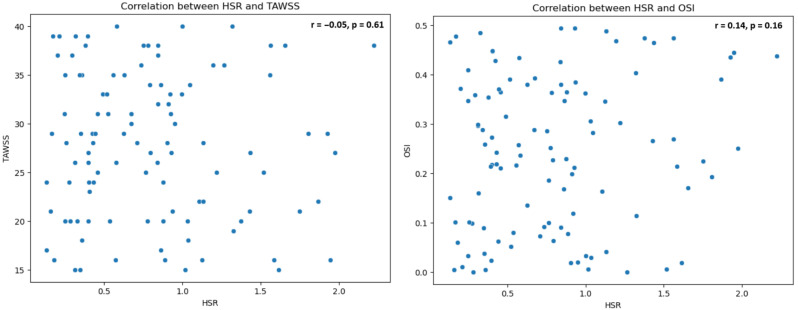
Scatter plots for HSR vs. TAWSS and HSR vs. *OSI*: HSR: Hyperemic Stenosis Resistance; TAWSS: Time-Averaged Wall Shear Stress; *OSI*: Oscillatory Shear Index.

The relationship between *cHSR* and the shear stress parameters TAWSS and *OSI* was evaluated. No significant correlation was found between *cHSR* and TAWSS (r = −0.05, *p* = 0.61), nor between *cHSR* and *OSI* (r = 0.14, *p* = 0.16) (Figure 6).

## 4. Discussion

### 4.1. Study Population

Our study included a cohort with suspected chronic coronary syndrome (CCS), encompassing a broad range of pretest probabilities and coronary lesion complexities. This diversity underscores the applicability of coronary computed tomography angiography (CCTA) as an initial diagnostic tool across varied patient profiles, aligning with recent guidelines and recommendations [2]. In a metanalysis conducted by Foy and al. [23], although CCTA did not significantly reduce mortality or cardiac hospitalizations, it was associated with a lower incidence of myocardial infarction, alongside an increase in invasive angiography, revascularizations, coronary artery disease diagnoses, and new prescriptions for aspirin and statins, compared to functional stress testing. This finding suggests CCTA’s role in identifying a higher burden of clinically significant CAD. Thus, traditional functional imaging—while valuable for ischemia detection—may have limited diagnostic performance in specific populations, such as those with three vessel disease and left main lesions, where anatomical assessment may offer superior guidance [12].

### 4.2. FFRCFD and FFR Correlation and Lumped Model Validation

**Figure 7:**
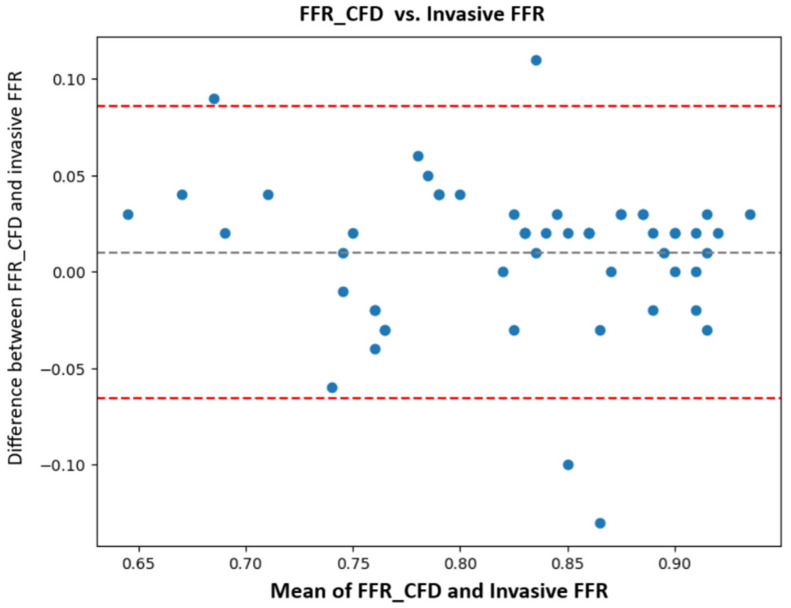
Bland-Altman analysis FFR_CFD_ vs. invasive FFR: computationally derived fractional flow reserve (FFR_CFD_); invasive fractional flow reserve (FFR).

In our study, FFR_CFD_ demonstrated excellent agreement with invasive FFR, with a Pearson correlation coefficient of 0.87, which is comparable to the results by Nørgaard et al. [24], who reported a correlation coefficient of 0.81, and Faulder et al. [25], who noted values between 0.82 and 0.85 in large, multicentric cohorts. Bland-Altman analysis in our study showed minimal bias, with most differences falling within ±0.10, affirming our model’s accuracy (Figure 7).

However, discrepancies between FFR_CFD_ and invasive FFR were observed in four cases, highlighting areas where further refinement of computational models may be beneficial. Two cases involved patients with a silent myocardial infarction (MI) in the territory of the coronary lesion. Prior research has shown that FFR values in post-MI patients can vary depending on the extent of myocardial viability. De Bruyne et al. demonstrated that viable myocardial tissue can maintain vasoreactivity even in infarcted regions, leading to variability in FFR measurements due to altered blood flow dynamics [26]. This suggests that in cases of silent MI, adjustments to computational models may be needed to accurately account for myocardial viability and regional perfusion changes.

Two other cases involved patients with a high coronary calcium burden, where segmentation challenges affected the precision of CCTA-derived models. High calcium scores can impair the accuracy of CCTA, impacting the segmentation process and introducing potential errors in model creation. Quality CCTA acquisition and advanced segmentation algorithms are critical in such scenarios, while the use of ultrahigh-resolution photon-counting CT may resolve this problem, with resolution levels approaching those of invasive coronary angiography [27].

The model’s high correlation likely benefits from the rigorous tuning of boundary conditions and patient-specific inputs such as flow adjusted to myocardial mass, which further improved alignment with invasive measures in respect to methods described previously [28,29].

### 4.3. cHSR Cutoff

Our analysis identified an optimal *cHSR* cutoff of 0.75 mmHg/cm·s^−1^ for predicting the necessity of revascularization, aligning closely with the findings of Meuwissen et al. [9], who proposed a hyperemic stenosis resistance (HSR) threshold of 0.8 mmHg/cm·s^−1^ in invasive measurements. However, this slight adjustment in cutoff in our study may result from the fidelity of our simulation methods. Despite promising preliminary evidence, larger studies are needed to substantiate this finding, especially to achieve the statistical power required to define an optimal *cHSR* threshold across diverse populations. Thus, prospective validations are crucial to reinforce the clinical applicability of *cHSR* as a complementary measure to FFR_CFD._

### 4.4. Diagnostic Performances of HSR, QFR, and FFRCFD

Both FFR_CFD_ and QFR displayed high diagnostic performance, consistent with prior studies (Table 4) such as Xu et al. [14]. and Agatsthi et al. [30], who reported sensitivities and specificities exceeding 80%. In our study, FFR_CFD_ and QFR achieved accuracies of 83% and 81%, respectively. However, *cHSR* demonstrated superior performance, with an accuracy of 96%, along with higher sensitivity and specificity than both FFR_CFD_ and QFR. This enhanced diagnostic capability of *cHSR* and aligns with the conclusions of Van de Hoef et al. [31], who noted that combined pressure and flow measurements can provide a more comprehensive understanding of coronary physiology than pressure indices alone. This combined approach for HSR appears to capture the hemodynamic impact of coronary stenosis more effectively, potentially refining patient selection for revascularization and addressing limitations inherent to pressure-only indices.

### 4.5. HSR and Shear Stress

Our investigation revealed no significant correlation between *cHSR* and shear stress parameters such as TAWSS and *OSI*, suggesting rather an intricate relationship between *cHSR* and shear stress. The literature on this topic is limited, particularly for computational indices, but some studies on invasive measures provide context. For instance, Puri et al. reported that pathological shear stress correlated with plaque vulnerability [32], while Wong et al. found moderate relationships between invasive indices and WSS parameters in specific lesion types [33]. The absence of a significant relationship in our study may indicate that while *cHSR* captures functional stenosis effects, it may not reflect local shear stress changes that influence plaque morphology and stability. This finding highlights the complexity of hemodynamic interactions, and the insufficient character of the current models to fully understand them.

**Table 4:** Summary of key studies evaluating the diagnostic performance and clinical benefit of computational fluid dynamics (CFD) and imaging-based coronary physiology indices.

Study	Index	Sample Size	Modality	Key Findings
**Nørgaard et al., 2014** [24]	FFR_CT	254	CCTA	Demonstrated high diagnostic accuracy of FFR_CT with invasive FFR as reference, sensitivity and specificity >80%.
**Westra et al., 2018** [34]	QFR	352	Angiography	QFR was highly accurate in predicting ischemia with FFR reference; achieved >85% accuracy.
**Xu et al., 2021** [14] **(FAVOR III China)**	QFR	3825	Angiography	Showed significant reduction in MACE over 1 year for QFR-guided PCI vs. standard angiography-guided PCI.
**Song et al., 2022** [35] **(2-Year FAVOR III)**	QFR	3825	Angiography	Demonstrated durability of QFR-guided PCI benefits with reduced MACE over 2 years compared to angiography guidance.
**Driessen et al., 2019** [36]	FFR_CT	208	CCTA	FFRCT showed higher diagnostic performance than standard coronary CTA, SPECT, and PET for vessel-specific ischemia.
**Koo et al., 2011** [37]	FFR_CT	103	CCTA	Accuracy, sensitivity, specificity, positive predictive value, and negative predictive value were 84.3%, 87.9%, 82.2%, 73.9%, 92.2%, respectively, for FFR_CT.

FFRCT: Fractional Flow Reserve derived from Coronary Computed Tomography Angiography; QFR: Quantitative Flow Ratio; CCTA: Coronary Computed Tomography Angiography; PCI: Percutaneous Coronary Intervention; MACE: Major Adverse Cardiac Events; SPECT: Single Photon Emission Computed Tomography; PET: Positron Emission Tomography.

## 5. Limitations

This study has several limitations. First, the retrospective design introduces potential selection biases, and the relatively small sample size may limit the generalizability of findings to larger populations. Additionally, the computational power and time required for 3D flow simulations pose challenges, as these processes are resource consuming and not yet feasible for routine clinical application. The study relied on retrospective acquisition protocols for CCTA, which increased cumulative radiation exposure for patients. Furthermore, the simulation models assume rigid arterial walls and omit arterial motion, which may not fully represent the dynamic nature of coronary arteries in vivo. Future work with larger sample sizes and advanced computational models may help overcome these limitations and increase the applicability of computational hyperemic stenosis resistance (*cHSR*) as a reliable diagnostic tool.

## 6. Conclusions

**Figure 8:**
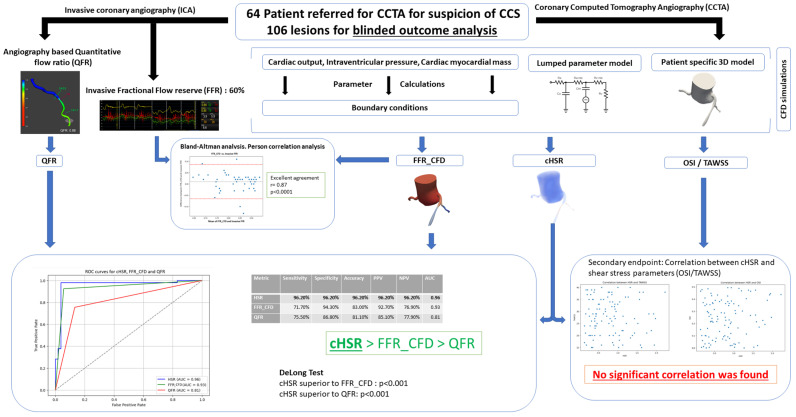
Central illustration: CCS: Chronic Coronary Syndrome; CCTA: Coronary Computed Tomography Angiography; ICA: Invasive Coronary Angiography; QFR: Quantitative Flow Ratio; FFR: Fractional Flow Reserve; FFR_CFD_: Fractional Flow Reserve derived from Computational Fluid Dynamics; *cHSR*: Computational Hyperemic Stenosis Resistance; *OSI*: Oscillatory Shear Index; TAWSS: Time-Averaged Wall Shear Stress; CFD: Computational Fluid Dynamics; AUC: Area Under the Curve; PPV: Positive Predictive Value; NPV: Negative Predictive Value.

Our study (Figure 8: Central illustration) demonstrates the promising diagnostic potential of a new computational index (*cHSR*), showing superior performance compared to traditional imaging-based indices. The computational model used in this study produced values of FFR_CFD_ with strong agreement with invasive FFR. These findings support the integration of computational methods into non-invasive imaging to improve decision-making in CCS management. Future advancements in automation and machine learning could enhance its clinical viability, potentially transforming how CCS is assessed and treated.

## Figures and Tables

**Table 1:** Baseline Characteristics of the Study Population.

Characteristic	Mean +/− (SD) or Percentage (%)
**Total Patients**	*n* = 64
**Age (years)**	67.8 ± 9.2
**Sex Ratio**	3 Male:1 Female
**Hypertension (HTN)**	62% (*n* = 40)
**Diabetes Mellitus (DM)**	39% (*n* = 25)
**Smoking History**	30% (*n* = 19)
**Dyslipidemia**	40% (*n* = 26)
**Obesity**	34% (*n* = 22)
**Chronic Kidney Disease (CKD)**	20% (*n* = 13)
**Pre-test Probability**	24.5 ± 14.8
**Calcium Score (CAC) (Agatston)**	113.1 ± 102.4

CAD: Coronary Artery Disease; HTN: Hypertension; DM: Diabetes Mellitus; CKD: Chronic Kidney Disease; CAC: Coronary Artery Calcium score.

**Table 2:** Lesion Characteristics.

Lesion Location	Mean +/− (SD) or Percentage (%)
**Total lesions**	*n* = 106
**LAD**	51 (49%)
**LCx**	26 (26%)
**RCA**	23 (22%)
**Left Main (LM)**	3 (3%)
**QCA (% Stenosis)**	55.3 ± 11.2
**Stenosis Length (mm)**	18.0 ± 7.4

LAD: Left Anterior Descending artery; LCx: Left Circumflex artery; RCA: Right Coronary Artery; LM: Left Main artery; QCA: Quantitative Coronary Angiography.

**Table 3:** Diagnostic Performance Metrics of *cHSR*, FFR_CFD_, and QFR.

Metric	Sensitivity	Specificity	Accuracy	PPV	NPV	AUC
***cHSR***	96.20%	96.20%	96.20%	96.20%	96.20%	0.96
**FFR_CFD_**	71.70%	94.30%	83.00%	92.70%	76.90%	0.93
**QFR**	75.50%	86.80%	81.10%	85.10%	77.90%	0.81

*cHSR*: Computational Hyperemic Stenosis Resistance; FFR_CFD_: Fractional Flow Reserve computed from Computational Fluid Dynamics; QFR: Quantitative Flow Ratio; PPV: Positive Predictive Value; NPV: Negative Predictive Value; AUC: Area Under the Curve.

## Data Availability

The datasets used and analyzed during the current study are available from the corresponding author upon reasonable request. Due to patient confidentiality and ethical restrictions imposed by the Institutional Review Board, the raw data cannot be publicly shared. However, de-identified data relevant to the findings of this study may be provided upon request, subject to institutional approval and compliance with data protection regulations.

## Supplementary Materials

The following supporting information can be downloaded at: https://www.mdpi.com/article/10.3390/jcm14207270/s1, Refs. [20,21,38,39,40,41,42,43,44] are cited in Supplementary Materials.
