# Supplementary material for: Performance of a Novel Computational Hyperemic Resistance Index Derived from Cardiac CT in Coronary Chronic Syndromes
Source: J Clin Med. 2025 Oct 15;14(20):7270. doi: 10.3390/jcm14207270 (PMC12565267; doi:10.3390/jcm14207270)
Supplement: Supplementary file 1 [file jcm-14-07270-s001.zip › jcm-3927583-supplementary.pdf]

Supplementary Methods

Patient-Specific 3D Model Elaboration

The 3D model construction for this study focused on representing the ascending aorta and the main branches of the coronary tree. Each coronary artery was modelled up to its first major bifurcation (expect for the left main bifurcation giving anterior descending and left circumflex). All arteries extended for additional 15 mm beyond a lesion if possible.

Segmentation Process

Segmentation was performed using an automatic machine learning (ML) algorithm integrated with SimVascular [1]. This segmentation approach allowed accurate identification of the coronary lumen, even in cases with high calcification. The segmentation process began with the selection of paths along each vessel's centerline. Paths were created using the "Control Points" feature in SimVascular, enabling alignment with the center of the coronary lumen. Paths were smoothed using Fourier smoothing to ensure a straight, consistent course through each centerline. Following path planning, 2D segmentations were generated along each path and then lofted to create the vessel geometry.

Meshing and Element Sizing

A radius-based meshing approach was applied to each vessel, with element sizes set at 0.03 mm to allow for a finer mesh along smaller vessels and complex curved segments. This method ensured sufficient detailing, particularly near regions of bifurcation and stenosis. By adhering to these meshing protocols, the final 3D model achieved the necessary balance between computational efficiency and accuracy in simulating coronary blood flow. A meshing independence test was realized to ensure less than 1.2% of the relative pressure fields cyclic variation between cycles at the inlet and outlets.

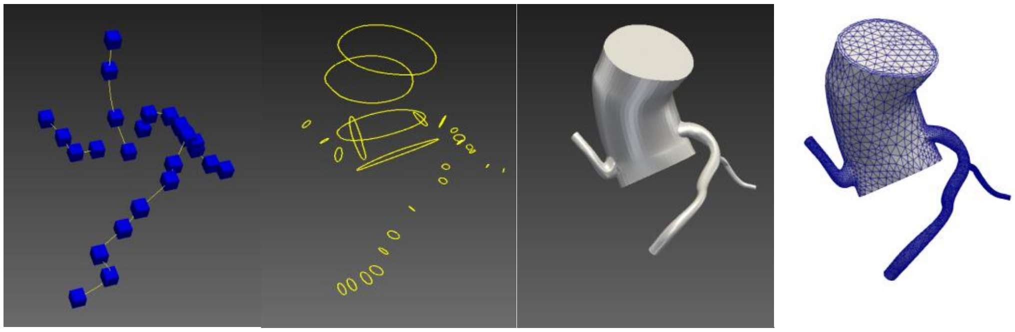

Figure S1. Steps to create a patient-specific 3D model of the vascular system. From left to right: (1) Extraction of vessel centerlines from imaging data, providing a simplified representation of vascular geometry. (2) Segmentation using machine learning algorithms to identify and delineate vessel boundaries. (3) Lofting process to construct a smooth 3D model of the vessels based on the segmented cross-sections. (4) Meshing of the 3D model for computational simulations, ensuring proper resolution and accuracy for analysis.

Simulation model parameters

Input parameters sources

To achieve accurate and patient-specific simulations, we gathered essential input data from multiple sources. Each parameter was tailored to match the individual patient's physiological characteristics using both non-invasive and invasive measurements.

- **Aortic inflow rate** was derived from MRI phase contrast data of a reference subject with similar Body Surface Area (BSA) to the patient. This reference flow profile was scaled to fit each patient's stroke volume and systolic ejection time, which we extracted from Cardiac CT volumetry measures. Myocardial mass was also calculated accordingly, and a perfusion percentage was allocated to each artery based on visual inspection and coronary dominance.
- **Patient-specific systolic and diastolic pressures** were obtained from invasive blood pressure measurements. These values provide the necessary inputs for modeling arterial pressure at various stages of the cardiac cycle. Noninvasive measurements can be a viable alternative in most of cases.
- **Intramyocardial pressure (P_{im})** was modeled using an invasive left ventricular pressure curve obtained from healthy subjects. To adapt this reference pressure curve to each patient, we scaled it according to the patient's specific aortic pressure measurements. This scaling ensures that P_{im} reflects the patient's myocardial loading conditions while preserving the temporal profile of left ventricular pressure changes. For modelling the right coronary arteries P_{im}, a scaling of 0.33-0.4 was applied to simulate the lower pressure levels in the right ventricle.

Aortic Outflow Model

For the aortic outflow, a classic RCR Windkessel model was considered, which incorporates three elements: proximal resistance (Rp), compliance (C), and distal resistance (Rd). These components collectively represent the systemic arterial system's resistance and elasticity. At baseline, we calculated the total systemic resistance from the patient's mean arterial pressure and cardiac output. The total resistance was distributed into Rp and Rd in a ratio of 1:9, emphasizing the higher resistance contribution of the distal microcirculatory beds. Compliance was initially set to 0.001 cm^5/dyne, ensuring a physiologically realistic systolic-diastolic pressure waveform with an amplitude in a reasonable range (40-60 mmHg).

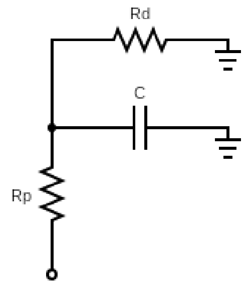

Figure S2.Schematic representation of the RCR (Resistance-Capacitance-Resistance) Windkessel model, showing proximal resistance (Rp), distal resistance (Rd), and compliance (C) used to simulate vascular impedance and flow dynamics.

Under stress conditions, cardiac output was increased by 300-350%, achieved through a combination of a 200-250% rise in stroke volume and a 120-160% increase in heart rate. The raised cardiac output necessitated recalibration of systemic resistance, targeting an increase by 140-150% of the mean aortic pressure. To maintain realistic pulsatile pressure waveforms, aortic compliance was reduced by 60% to reflect the stiffer arterial conditions seen in stress. A second process of tuning in 5% steps was performed to ensure a smooth pressure decay and reasonable systolic-diastolic pressure differences. These adaptations allowed the aortic outflow model to accurately respect the hemodynamic shifts associated with increased workload.

Equation S1

$$R_{tot} = \frac{Mean\ pressure}{Cardiac\ output}$$

Lumped Parameter Model for Coronary Outflows

The coronary circulation was represented using a lumped parameter network [2] comprising resistances (R_a, $R_{a\text{-}micro}$, Rv), capacitances (C_a, C_{im}), and an intramyocardial pressure (P_{im}):

- **Resistances (R_a, $R_{a\text{-}micro}$, R_v):** These represent the flow impedances in different regions of the coronary vasculature. R_a models proximal resistance in larger arteries, $R_{a\text{-}micro}$ corresponds to resistance within the microcirculation, and R_v accounts for resistance in the venous outflow. We considered a zero value of $R_{v\text{-}micro}$, which had little effect on the output as reported previously [3].
- **Capacitances (Ca, Cim):** These simulate the ability of vessels to store blood. Ca models arterial compliance, while Cim reflects the compliance within the myocardium, influenced by myocardial contraction. A ratio of 0.11:0.89 was constantly used in our simulations, as previously reported [3].
- **Intramyocardial Pressure (Pim):** This pressure, derived from left ventricular pressure curves, captures the compressive forces exerted on coronary vessels during systole and diastole. It introduces a key feature of coronary physiology: the out-of-phase relationship where flow is predominantly diastolic.

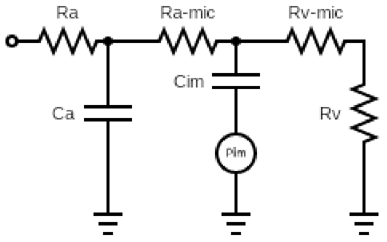

Figure S3 Lumped parameter model of coronary circulation, depicting proximal arterial resistance (Ra), microvascular arterial resistance (Ra-mic), microvascular venous resistance (Rv-mic), venous resistance (Rv), arterial compliance (Ca), microvascular compliance

This model was able to reproduce the unique out-of-phase flow characteristics of coronary arteries, where flow is predominantly diastolic. The total coronary flow was set at 4-5% of cardiac output, consistent with physiological coronary perfusion. The flow distribution to each artery was adjusted to the patient's myocardial mass based on previous studies [4–8]. Total coronary resistance (using the same equation as

equation 1 for coronary circulation) was found as 24 times the systemic resistance to account for the higher resistance characteristic of coronary microcirculation. At rest, this resistance was distributed among the components as follows: R_a (32%), $R_{a\text{-}micro}$ (52%), and R_v (16%) as reported in previous studies [3,9]. Intramyocardial pressure (P_{im}), derived from left ventricular pressure curves, was scaled to match patient-specific pressures. A scaling of 0.33 of P_{im} for the right coronary arteries (RCA) was respected, ensuring accurate external compression by the right ventricle.

The distribution of coronary resistance and compliance among outlets was guided by physiological principles and geometric considerations. Resistance was allocated based on the outlet's distal cross-sectional area following a generalization of Murray's Law, which posits that resistance is inversely proportional to the radius raised to the power of 2.6. Similarly, compliance distribution was proportionate to the outlet area.

Equation S2

$$R_i = R_{total} \cdot \frac{r_i^{-2.6}}{\sum_j r_j^{-2.6}}$$

Where:

- R_i is the resistance assigned to the outlet i.
- R_{total} is the total coronary resistance.
- r_i is the radius of the outlet.
- The denominator $\sum_j r_j^{-2.6}$ is the summation of all outlet radii raised to the power of -2.6

Equation S3

$$C_i = C_{total} \cdot \frac{A_i}{\sum_j A_j}$$

Where:

- C_i is the compliance assigned to the outlet i.
- C_{total} is the total coronary compliance.
- A_i is the cross-sectional area of outlet i.

Under stress, the coronary model underwent several adjustments to accommodate increased cardiac workload. While the total coronary flow maintained its proportion of 4-5% of the elevated cardiac output, the total coronary resistance was recalculated to align with the higher cardiac output and redistributed among the resistance components (R_a, $R_{a\text{-}micro}$, R_v) using a different ratio (45%, 33%, 22%). This distribution strategy preserved coronary flow in regions with non-significant (<30%) or no stenosis while recreating the physiological pressure drop observed in the presence of a more significant one. Coronary compliance was increased by 100% to accommodate the higher coronary flow demands, and tuned in 10% steps to achieve smooth pressure curves. Additionally, P_{im} was scaled to higher levels to match increase in systolic aortic pressure. Distribution of resistance and compliance to each coronary outlet respected the same reasoning as at rest.

Table S1. Initial values and tuning process

PARAMETER	**INITIAL VALUE OR CALCULATION METHOD**	**STRESS ADJUSTMENTS**	**TUNING PROCESS/ TARGET**
AORTIC RESISTANCE (R)	Calculated using mean aortic pressure and cardiac output, split as Rp: Rd in 1:9 ratio.	Recalculated to reflect 140-150% increase in mean aortic pressure.	Automatically scaled with cardiac output to match increased systemic pressures.
AORTIC COMPLIANCE (C)	Set to 0.001 cm^5/dyne to match physiological systolic-diastolic pressure waveforms.	Reduced by 60% to account for arterial stiffening during stress.	Adjusted iteratively in 5% steps to maintain reasonable pressure decay and systolic-diastolic amplitude.
TOTAL CORONARY FLOW	Fixed at 4-5% of cardiac output, normalized to myocardial mass.	Increased proportionally with cardiac output to maintain 4-5% coronary flow distribution under stress.	No additional tuning; scaled directly with cardiac output.
TOTAL CORONARY RESISTANCE (R_{COR})	Calculated as 24x systemic resistance, distributed among Ra (32%), Ra-micro (52%), and Rv (16%).	Recalculated based on increased cardiac output, distributed among Ra (45%), Ra-micro (33%), and Rv (22%).	Adjusted to achieve accurate pressure-flow curves.
R_A (PROXIMAL RESISTANCE)	32% of total coronary resistance, calculated based on outlet diameters using Murray's Law ($r^{2.6}$).	45% of total coronary resistance; adjusted to ensure physiological stress flow drop in significant lesions	Adjusted to achieve accurate pressure-flow curves.
$R_{A\text{-}MICRO}$ (MICROCIRCULATORY RESISTANCE)	52% of total coronary resistance, reflecting capillary network characteristics.	33% of total coronary resistance	Adjusted to achieve accurate pressure-flow curves.
R_V (VENOUS RESISTANCE)	16% of total coronary resistance, representing venous return properties.	22% of total coronary resistance	Adjusted to achieve accurate pressure-flow curves.
CORONARY COMPLIANCE (C)	Distributed as C_a (11%) and C_{im} (89%) based on outlet area.	Increased by 100% to accommodate higher coronary flows under stress conditions.	Adjusted iteratively in 10% steps to match physiological pressure curves at each coronary outlet.
INTRAMYOCARDIAL PRESSURE (PIM)	Derived from left ventricular pressure curve, scaled 0.33 for RCA.	Scaled to meet aortic systolic pressure increase	No further tuning

Reduced order models and tuning process

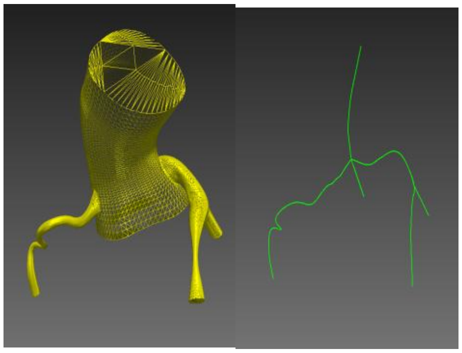

Figure S4. From 3D model to 1D centreline for reduced order simulations

The 1D model is generated by extracting vascular centerlines from a 3D model, discretized into segments for finite element analysis of flow and pressure. Lumped parameter models represent the aortic and coronary trees using resistances, compliances, and intramyocardial pressure. Iterative simulations are conducted to tune these parameters, including resistance distributions and compliance values, through trial-and-error adjustments, ensuring alignment with defined boundary conditions and flow dynamics (Table S1. Initial values and tuning process).

Solver parameters description

In simulations, wall properties assume rigid vessel walls with no-slip and no-penetration boundary conditions applied to blood flow. While this approach simplifies the model, more advanced simulations can incorporate wall deformation through fluid-structure interaction (FSI) for greater realism, this approach in the other hand, increase computational resources necessary to perform simulations. Solver parameters were configured to ensure computational stability. The simulations were set to run for 4000-9000 timesteps with a timestep size of 0.001 seconds, adjusted to the patient heart rate of beats per minute (cardiac cycle duration in seconds). Results were saved every 20 timesteps, with linear solver settings (Step Construction = 5 and Maximum Iterations = 10) optimized for coronary simulations. Blood was modeled with a viscosity of 0.04 poise and a density of 1.06 g/cm³, which are standard physiological values. These settings ensure the fidelity of the hemodynamic simulation while maintaining computational efficiency.

References

1. Maher G, Parker D, Wilson N, Marsden A. Neural Network Vessel Lumen Regression for Automated Lumen Cross-Section Segmentation in Cardiovascular Image-Based Modeling. *Cardiovasc Eng Tech*. 2020;11(6):621-635. doi:10.1007/s13239-020-00497-5

2. Mantero S, Pietrabissa R, Fumero R. The coronary bed and its role in the cardiovascular system: a review and an introductory single-branch model. *Journal of Biomedical Engineering*. 1992;14(2):109-116. doi:10.1016/0141-5425(92)90015-D

3. Kim HJ, Vignon-Clementel IE, Coogan JS, Figueroa CA, Jansen KE, Taylor CA. Patient-Specific Modeling of Blood Flow and Pressure in Human Coronary Arteries. *Ann Biomed Eng*. 2010;38(10):3195-3209. doi:10.1007/s10439-010-0083-6

4. Choi JH, Kim E, Kim HY, Lee SH, Kim SM. Allometric scaling patterns among the human coronary artery tree, myocardial mass, and coronary artery flow. *Physiological Reports*. 2020;8(14):e14514. doi:10.14814/phy2.14514

5. Kassab G, Finet G. Anatomy and function relation in the coronary tree: from bifurcations to myocardial flow and mass. doi:10.4244/EIJV11SVA3

6. Choy JS, Kassab GS. Scaling of Myocardial Mass to Flow and Morphometry of Coronary Arteries. *J Appl Physiol*. 2008;104(5):1281-1286. doi:10.1152/japplphysiol.01261.2007

7. Murai T, van de Hoef TP, van den Boogert TPW, et al. Quantification of Myocardial Mass Subtended by a Coronary Stenosis Using Intracoronary Physiology. *Circulation: Cardiovascular Interventions*. 2019;12(8):e007322. doi:10.1161/CIRCINTERVENTIONS.118.007322

8. Van Rosendael SE, Van Rosendael AR, Kuneman JH, et al. Coronary Volume to Left Ventricular Mass Ratio in Patients With Hypertension. *The American Journal of Cardiology*. 2023;199:100-109. doi:10.1016/j.amjcard.2023.04.025

9. Ghorbanniahassankiadeh A, Marks DS, LaDisa JF Jr. Correlation of Computational Instantaneous Wave-Free Ratio With Fractional Flow Reserve for Intermediate Multivessel Coronary Disease. *Journal of Biomechanical Engineering*. 2021;143(051011). doi:10.1115/1.4049746
